# Bone finds and their medicolegal examination: a study from Hesse, Germany

**DOI:** 10.1007/s12024-023-00599-1

**Published:** 2023-04-17

**Authors:** T. E. N. Ohlwärther, F. Holz, K. Edler, S. C. Kölzer, E. Reuss, M. A. Verhoff, C. G. Birngruber

**Affiliations:** 1 Institute of Legal Medicine, University Hospital Frankfurt, Goethe-University, Kennedyallee 104, Frankfurt am Main, 60596 Germany; 2 Institute of Legal Medicine, University Hospital Gießen and Marburg, Justus-Liebig-University, Gießen, Germany

**Keywords:** Forensic osteology, Anthropology, Postmortem interval, Bony injuries, Identification

## Abstract

Bones found by chance can be of great criminal or historical interest. The nature of their appraisal depends on the individual case, the locally effective legislation and the available resources. To assess whether a find is relevant with respect to criminal investigation, the circumstances of the find and the results of the forensic examination carried out by trained personnel must be considered. The aim of this study was to obtain an overview of the circumstances and nature of the finds as well as the results of the subsequent expert opinions by evaluating bone finds from the federal state of Hesse, Germany. For this purpose, over a 10-year period from 2011 to 2020, all bone finds examined at the Institutes of Legal Medicine in Gießen and Frankfurt am Main, Germany, were evaluated retrospectively with regard to the locations and circumstances of the finds, their nature (human or non-human), the postmortem interval, possible traces of violent impact and the results of further examinations. Of the 288 bone finds evaluated, 38.2% were found in forests, meadows and parks. In 50.7%, the finds contained human bones, of which 37.0% had a forensically relevant postmortem interval of 50 years or less. Evidence of trauma was described in 77.4% of the human bone cases: postmortem damage in 78.8%, peri-mortem injury in 9.7% and ante-mortem injury in 11.5%. DNA examinations were performed in 40.4% of the human bone finds. They yielded STR profiles in 81.3%, leading to a definite identification in 35.4%. Among the non-human bones sent in, the most common were bones from pigs (23.4%), deer (18.1%), cattle (16.4%), roe deer (11.7%) and sheep (11.7%). The macroscopic examination is the first step of the forensic-osteological evaluation and sets the course for further examinations or investigations. DNA examinations are of great importance for the reliable identification of human bones. They were responsible for 70.8% of successful identifications.

## Introduction

With an area of 21,115 km^2^ and a population of 6.4 million, Hesse is the fifth largest of the sixteen German federal states [1]. It is divided into nine regional court districts. The responsibility for these districts falls to two different Institutes of Legal Medicine: Gießen and Frankfurt am Main. Service for the northern half of Hesse, with the regional court districts of Gießen, Fulda, Kassel, Limburg and Marburg, is provided by the Institute of Legal Medicine in Gießen, while the Institute of Legal Medicine in Frankfurt am Main is responsible for the southern half with the regional court districts of Darmstadt, Frankfurt am Main, Hanau and Wiesbaden. The area covered by the Institute in Gießen is largely rural, while that of the Institute in Frankfurt am Main includes the Rhine-Main metropolitan area.

If skeletons, bones, bone fragments or alleged bones are found by chance outside of archaeological excavation sites in Hesse and reported to the police, the investigating authorities usually commission an expert opinion. The find then goes to the regionally responsible Institute of Legal Medicine, where a medicolegal or forensic-osteological examination is performed.

The assessment is carried out according to a specific regimen aiming at answering questions relevant to the case [2, 3]: If the find is a bone, the question of human origin should be addressed first [4]. If this is not confirmed, a determination of the animal species and a description of any injury marks or damage should be made. In the case of non-human bones, there is usually no further need for action by the investigating authorities.

If human bones are involved, further examination procedures follow, regarding the postmortem interval (PMI) [5–10], pre- or peri-mortem traces of injury or postmortem defects [11–13] and findings indicating or assuring identity [14–21]. The results of the examination should be interpreted and presented in a written report to support the authorities in their further investigations [3, 22].

The objective of the presented study was to portray the phenomenology of bone finds examined by both Institutes of Legal Medicine in Hesse, Germany, by means of a retrospective systematic analysis, and to put them into a national and international context.

## Material and methods

For a period of 10 years (2011–2020), all bone finds examined by the Institutes of Legal Medicine in Gießen and Frankfurt am Main, Hesse, Germany, were analysed.

All analogue and digital archived records of forensic-osteological examinations in both institutes were reviewed. For further analysis, examinations of bones after planned exhumations or the clearance of ossuaries were excluded [23]. Furthermore, finds that, by definition, were not skeletons or bones but (still) corpses or parts thereof [24, 25] were not considered.

Information was then evaluated regarding sites and discoverers, the nature of the bones, the postmortem interval, possible traces of violence and the results of further investigations including whether the bones could be assigned to a specific person.

Consistency in the way the examinations were conducted can be assumed. Three of the authors have been working in both Institutes of Legal Medicine, ensuring a harmonized assessment. At any point, the examinations were carried out based on the so-called “generally accepted state of the art”.

## Results

During the 10-year study period, 288 forensic-osteological examinations were performed by the two Institutes of Legal Medicine. In Frankfurt am Main, 159 cases were processed, and in Gießen 129.

### Caseload

**Fig. 1:**
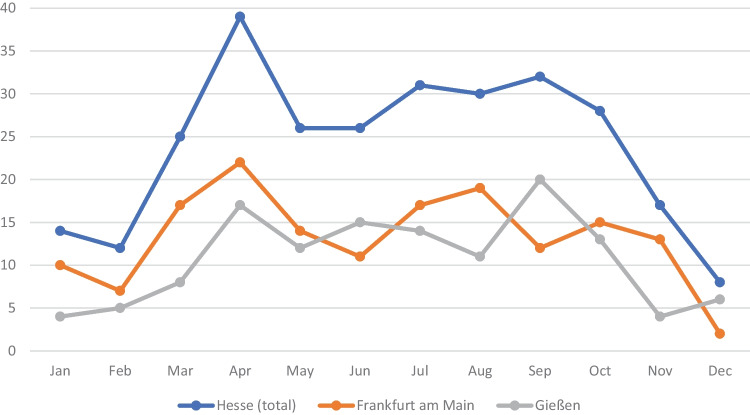
Monthly distribution of the bone finds, cumulative numbers from 2011 to 2020 (*N* = 288)

The number of examinations per year varied between 18 and 48 and was unevenly distributed over the individual months of the year (Fig. 1). During the months of summer (April to September) with 63.9% (*n* = 184), notable more finds were reported than in the winter. Most bone finds occurred in April. On average, 2.4 bone finds per month or 28.8 per year were assessed during the study period.

### Locations and finders

**Fig. 2:**
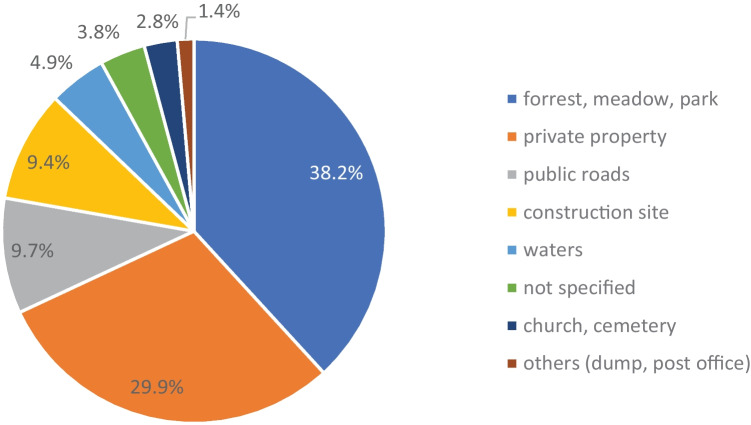
Sites of the finds (*N* = 288)

**Fig. 3:**
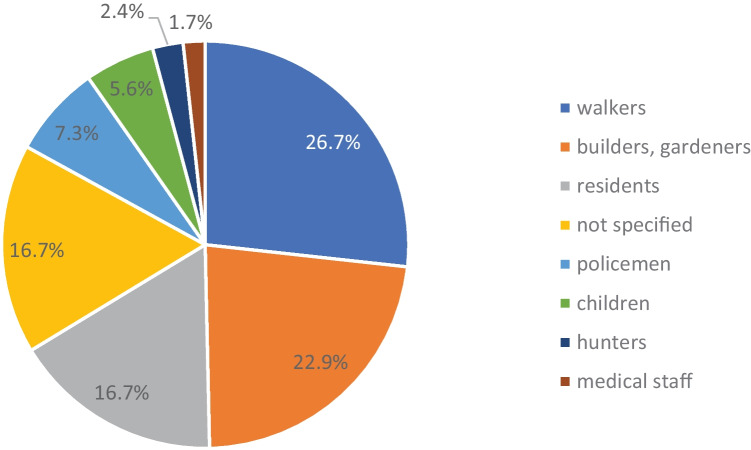
Finders (*N* = 288)

Regarding locations and discoverers, there were no significant differences between the rather rural area covered by the Institute of Legal Medicine in Gießen and the rather urban areas managed by the Institute of Legal Medicine in Frankfurt am Main. More than one-third of the bone finds occurred in forests, meadows or large parks, followed by private property, public roads and construction sites (Fig. 2). The bones found in cemeteries and churches were finds that could not be assigned to a regular burial and were found, for example, above ground between intact graves or on the steps of the church. More than one in four bone finds (26.7%; *n* = 77) were reported by walkers or hikers, often while walking their dogs. Workers (construction) or gardeners reported 22.9% (*n* = 66) of the finds. Among the finders were also police officers, hunters and medical personnel who found bones during their professional activities (Fig. 3).

### Nature of bones

In nearly half of the cases, the find contained animal bones exclusively (48.6%; *n* = 140) and somewhat less frequently human bones exclusively (43.8%; *n* = 126). In 6.9% (*n* = 20), human and animal bones were found together. In two of the cases (0.7%), the finds were not even bones: a piece of rolled linoleum flooring and a plastic bone were found and sent in for examination (Table 1).

**Table 1:** Nature of cases (*N* = 288)

**Period**	**Human bones**	**Animal bones**	**Human and animal**	**No bones**	**Sum**
2011	6	10	2	0	18
2012	13	7	0	0	20
2013	17	13	2	0	32
2014	10	12	1	0	23
2015	14	11	3	0	28
2016	12	13	3	0	28
2017	15	10	3	0	28
2018	13	31	3	1	48
2019	12	10	1	0	23
2020	14	23	2	1	40
2011–2020	126	140	20	2	288

### Type of bones

**Fig. 4:**
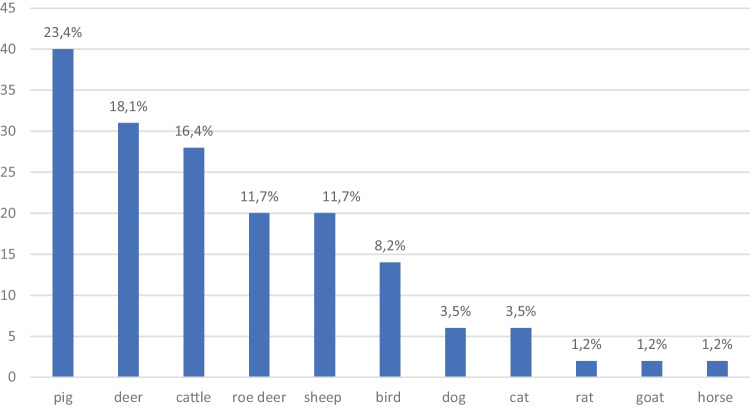
Species of the animal bones

**Fig. 5:**
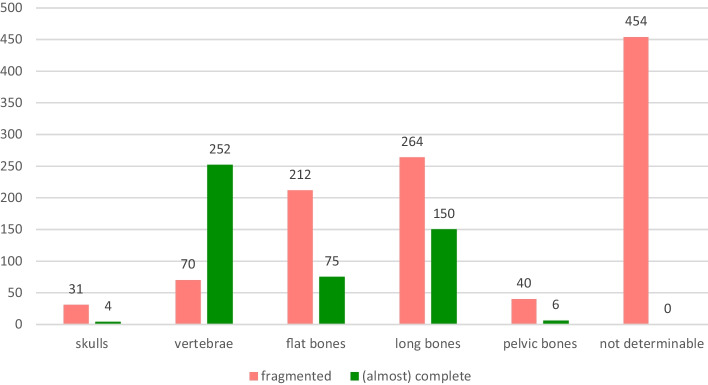
Animal bones, types and conditions

**Fig. 6:**
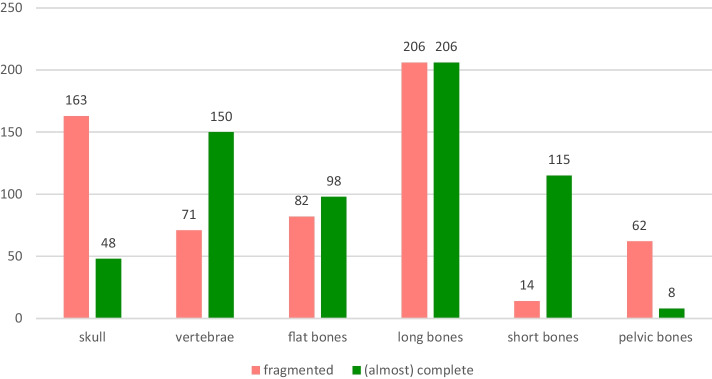
Human bones, types and conditions

A total of 13 (nearly) complete human skeletons and 2781 other bones were examined. Of the other bones, 1112 were (nearly) complete and 1669 were fragmented. Of all bones and bone fragments examined, 43.9% (*n* = 1223) were of human origin. Among the animal bones recovered, 23.4% (*n* = 40) were pig bones followed by deer bones with 18.1% (*n* = 31) and cattle bones with 16.4% (*n* = 28). Bones of roe deer and sheep were each found in 11.7% (*n* = 20) of the cases (Fig. 4). Among the human bones, skull or cranial parts were present in 17.3% (*n* = 211); among the animal bones, skulls or parts thereof were present in only 2.2% (*n* = 35) (Figs. 5 and 6).

### Postmortem interval

Macroscopic assessment of the postmortem interval was performed in all cases of human bone finds. Additional examinations were performed in 43.2% (*n* = 63) of these cases. Among these were UV florescence testing on the fresh saw surface (60.3%), examination of bone powder with luminol (34.9%; *n* = 38) and radiocarbon dating (4.8%; *n* = 3).

There were equal numbers of cases with a forensically relevant postmortem interval (PMI ≤ 50 years) and cases with a PMI of 50 years or more (37.0%; *n* = 54 each). In the remaining 26.0% (*n* = 38) of the human bone finds, no reliable statement could be made regarding the PMI due to a longer period of decay outdoors and above ground. Radiocarbon dating was used to date bone finds to the fifteenth, seventeenth and eighteenth centuries.

### Signs of trauma and damage

The majority of all bone finds showed traces of ante-, peri- or postmortem trauma. Postmortem damage was most frequent (58.3%; *n* = 168), of which 19.6% (*n* = 33) were specified as animal damage and 13.1% (*n* = 22) as recovery damage. Peri-mortem injuries were present in 16.0% (*n* = 46) of all cases, of which 67.4% (*n* = 31) were found as butchery marks, exclusively on animal bones. Ante-mortem injuries were described in 4.9% (*n* = 14) of all cases and exclusively for human bones.

Lesions were described in 77.4% (*n* = 113) of the cases containing human bones. These were postmortem damage in 78.8% (*n* = 89), peri-mortem injuries (from blunt force, gunshot, sharp force) in 9.7% (*n* = 11) and ante-mortem findings (healed fractures, surgical procedures, inflammatory changes) in 11.5% (*n* = 13).

### Identification

#### Morphological methods

The examination protocol used in both Hessian Institutes of Forensic Medicine includes the determination of a biological profile for each human bone find, in order to obtain information regarding the unknown identity. In addition, a detailed dental status was obtained in 32.2% (*n* = 47) of the human bone cases.

Definite identifications were made in six cases by the dental status and in one case by craniofacial photografic superimposition.

#### Molecular genetic investigations

Molecular genetic analysis was used in 24.7% (*n* = 71) of all cases and in 40.4% (*n* = 59) of human bone cases. STR typing was successful in 81.3% (*n* = 48) of these cases. Of these, in 35.4%, i.e. in 17 cases, matching of these postmortem STR profiles with ante-mortem comparison profiles of missing persons or their biological relatives led to a definite identification.

## Discussion

Bone finds can be of considerable interest to, for example, law enforcement and historic preservation authorities. Depending on the applicable legal regulations and the circumstances of the individual case, the person making the discovery will report the find to the police, thus initiating subsequent investigations. The number of cases investigated in this study does not reliably include all bone finds made in Hesse. Bones may be correctly identified as animal by competent finders or incorrectly misidentified as non-human by laypersons and therefore not be reported. Other finders may deliberately not report bone finds for fear of delays and cost increases in construction projects, for example.

In Germany, bone finds reported to the police are usually examined in forensic-osteological examinations at the Institutes of Legal Medicine. Anthropological institutes that would conduct such an assessment are scarce and far apart [26, 27]. Anthropologists work sporadically at the Institutes of Legal Medicine in German-speaking countries, but mainly the examinations are performed by forensic physicians. In many federal states of Germany, acquiring the necessary expertise is part of the underlying specialty training in Legal Medicine. The processing of bone finds differs greatly from other countries, where forensic anthropology as an independent subject has a broader profile [28–35].

The presented evaluation of bone finds from Hesse, Germany, was intended to give an overview of these investigations, which in individual cases may be very consequential. Furthermore, it was supposed to show possible regional peculiarities in a (inter-) national comparison. The study was limited to cases with purely forensic-osteological examination. Cases in which the human remains were corpses in the sense of the applicable legislation were not considered, in contrast to other studies dealing with broader forensic-anthropological casework [30, 36]. Human remains that were corpses according to current law [24, 25] were examined in a forensic autopsy by two physicians in accordance with the German Code of Criminal Procedure (§ 87 StPO).

Publications dealing with the type and frequency of bone finds and their forensic-osteological or forensic-anthropological examination are rather rare.

### National data

At the national level, a comparison with data collected for the period 2011–2015 from a publication on “forensic anthropological bone finds” from Hamburg, Germany, seems appropriate [37].

The number of cases is only slightly lower with a significantly smaller size and population of Hamburg. The month in which the most frequent bone finds occurred was March; bone finds in the months of summer (58%) were more frequent than in the months of winter (40%). In 2%, the month of finding remained unknown in Hamburg. As a cause for the accumulation of finds in April, rising temperatures and increased activity of people outdoors can be assumed, both in terms of recreational activities and gardening as well as construction work.

In Hamburg, 50% of all bones were found during construction work or gardening. Here, the finds were reported in 32% by construction workers and in 13% by residents. In comparison, in Hesse, 22.9% were found by construction workers or gardeners and 16.7% by residents. The proportion of walkers or hikers reporting a bone find was 20% in Hamburg. This is below the 26.7% in Hesse, which could be due to the vast forest and meadow areas used for walks in Hesse in the rural areas. These are the areas in which 38.2% of all bone finds were made in Hesse, while the Hamburg study comes down to about 12.7% that were found in open nature (without water areas). It is worth mentioning that in one case in Hamburg, a bone find was forwarded to the Institute of Legal Medicine via the “lost and found” office.

In Hamburg, 47% of the finds contained human bones, in Hesse 50.7%. Mixed finds were present in 10% and 6.9% of the cases, respectively, and non-bony material was found in 1% and 0.7% of the cases. The Hamburg study does not contain any information on the animal species; the non-bony material was charcoal.

The proportion of bones with a forensically relevant postmortem interval was 37.0% of the human bone finds in Hesse and 28.7% in the Hamburg collective. Successful identifications were achieved in Hamburg in three cases via DNA examinations and in two cases by means of morphological examinations. In Hesse, identification was achieved by morphological methods in seven cases and by molecular biological methods in 17 cases.

Regarding signs of trauma and damage, in our study and the Hamburg study, the classification into pre-, peri- and postmortem was conducted according to scientifically sound definitions widely used in casework [38–40]: Premortem injuries show signs of healing; in contrast, peri-mortem injuries, which occur shortly before, at, or shortly after death, show no signs of healing; Postmortem damage refers to damage acquired long after death and presents fracture patterns compared to the fracture characteristics of an old, dry tree branch. Information given in the Hamburg study is limited to merely stating that ante-mortem injuries were described in some cases and peri-mortem injuries were rarely present.

### International data

For international comparisons of certain parameters, the literature includes data from Switzerland [41], the USA [42–47], South Africa [36, 48] and Australia [31, 49].

Due to differences in national legislation responsibilities and allocations and the resulting pre-selections, comparisons of case numbers are mostly pointless, and caution is necessary when comparing other parameters. An accumulation of bone finds in the summer half-year comparable to our data was found in Bern, Switzerland, and can be explained by increased recreational activities and a lack of snow cover during this time. In New Mexico, USA, there was no correlation with find frequency and season [41]. A study of bone finds from primarily the western and southern part of the USA reported more frequent finds towards the end of the year due to the hunting season [42].

In the Swiss study, the site of discovery was most frequently outdoors and in 37% of the cases in the mountains or forests [41]. In southern Australia [49], bones were most frequently found in developed land including building sites or residential areas (64.7%) and only in 14.7% in open fields, whereas in New South Wales [31], almost equal numbers were found in the city (48%) and in the bush (47%). In the collective from Gauteng, South Africa, the majority of cases were found in open fields, and one bone was taken from a “witch-doctor” [48]. Bones were most frequently found by hikers and climbers (50%) in the Swiss study and most frequently by outdoor recreationists (11.1%) in the Grisbaum study with incomplete data. The results of international studies overall match our findings with walkers as the most frequent finders (26.8%) and forests and meadows as the most frequent sites (38.2%).

In Hesse, Germany, 55.6% of all cases contained animal bones, in Switzerland 32.8% [41] and in Southern Australia 70% [49]. The numbers for investigations from the USA vary by region [50], ranging from 18.8% in California [43] to 23.6% in Tennessee [46] and 28% for FBI cases [42] to 50% in New York City [47]. From South Africa, only 1.6% animal bones are reported among cases investigated in Johannesburg [48]. As a unique feature of this study, it should be noted that the allocations only came from mortuaries and forensic pathology services and can be subsequently assumed to be pre-selected.

**Fig. 7:**
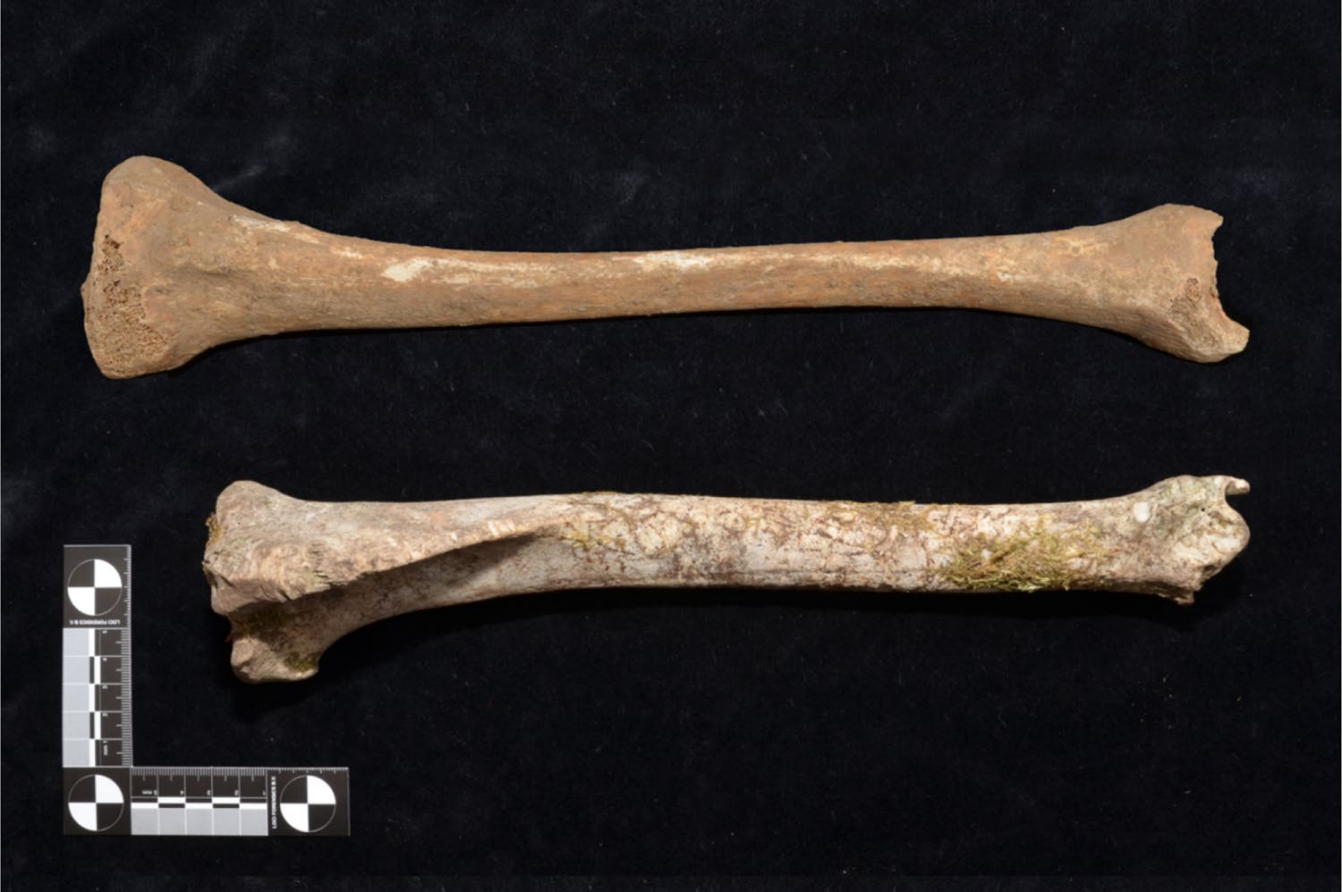
Left human tibia (top), right deer tibia (bottom)

The animal species of which the bones were most frequently sent for examination vary by region [49] and depend on three factors: the frequency of occurrence of an animal species locally, the resistance of the bones to environmental influences and the similarity to the human skeleton [50]. For North America, for example, a high similarity of skeletonized bear paws to the human hand skeleton has been described. The same is observed in Romania, as shown in a richly illustrated case report [51]. In Australia, the radius of a kangaroo shows great similarities to that of a human [49]. The most common animal bones found in Australia are vertebrae and long bones from sheep and cattle [49] and bones from sheep and kangaroos [31]. Donlon et al. [50] see a connection between found bones and local eating habits due to traces of slaughter presented on the bones. In their paper, they give a short outline of the animals most frequently eaten in Australia in the past decades. The idea that consumption habits of humans have an influence on the frequency of animal bone finds would match the results from Hesse: Pork bones were the most frequently found animal bones in the study collective. And pork is the most common type of meat found on German plates [52]. A most likely confusion with a human bone existed in Hesse for the deer tibia (Fig. 7) [53]. In 71.4% of the cases in which medical personnel or hunters, i.e. persons with special knowledge of the human or animal skeleton, mistook an animal bone for human and reported it to the police, it turned out to be the tibia of a deer or roe deer. The type of animal bone that was most rarely sent in for examination was the skull. This is surely due to the fact that the skull of an animal is easily distinguishable from a human skull, even for laypersons.

A forensically relevant PMI of 50 years or less was present in 37.0% of human bone finds in Hesse. In the Swiss study, a threshold of 60 years was set as the definition for cases of forensic interest. As a result, 37.9% of all bone findings were classified as forensic remains. Donlon reports that the majority of their cases (57%) from New South Wales, Australia, were recent, using a cut-off of 50 years for the PMI [31]. Simpson and Byard mention a part of 18% of their cases as “contemporary” [49]. In the USA, 35% of the reported cases from New Mexico even had a PMI of less than one week, but as Komar presents forensic anthropology casework, his study included more than only skeletonized remains [44]. The same applies for the work of Grisbaum and Ubelaker, who report a proportion of 66.2% of their FBI cases having a PMI of less than 10 years [42].

Signs of trauma were detected in 77.4% of all human bone cases in Hesse. Of these, 78.8% were postmortem, 9.7% peri-mortem and 11.5% ante-mortem. The authors from Switzerland report postmortem damage for all their forensic cases, peri-mortem trauma in 36.4% and ante-mortem trauma in 4.6% [41]. In cases from the Western Cape, South Africa, peri-mortem trauma was observed in 29% and ante-mortem trauma in 41% [36]. For New Mexico, USA, Komar gives the information that in 42% of the cases, no evidence of peri-mortem trauma was observed and that postmortem damage in the sense of animal activity was present in 46% of the cases [44]. Ubelaker reports a proportion of 15% of skeletal remains that showed evidence of scavenging by animals [45].

Cases from Hesse, where the identity of the bones could be clarified, were solved with molecular genetic investigation in 70.8% and with morphological methods in 29.2%. The team from Bern, Switzerland, used DNA analysis for 100% of their positive identifications [41]; in Gauteng, South Africa, four of the identifications were made through DNA confirmation and one case through the use of secondary identifiers [48]. Baliso’s study from South Africa [36] impressively demonstrates the importance of police investigation and the availability of comparative data for successful identification. While ante-mortem trauma was observed in 41% of decedents, the lack of medical records hindered the use of this information for identification. Nevertheless, positive identifications were reached for 37% of decedents. Of the cases with an indication of identity, 81% reached a positive identification. Of these cases, human remains were 12 times more likely to be identified.

## Conclusions

The professional examination of bones and skeletonized remains found by chance is mandatory to identify cases of forensic interest; regardless of whether this task is performed by forensic anthropologists or medicolegal experts. Information regarding human specificity, number of individuals, postmortem interval, type and aetiology of injuries and hints to identity can usually be provided by macroscopic examination. Depending on the circumstances of the individual case and available comparative material, macroscopic or molecular genetic examinations may be considered for reliable identification. When examining osseous remains, the objective should be to contribute to solving cases of missing persons and to reveal undiscovered unnatural deaths.

## Key points

1. The professional examination of bones found by incidence is mandatory to identify cases of forensic interest.
2. In Hesse, 50.7% of the finds contained bones of human origin. The most frequent animal bones sent in for examination derived from pigs, deer and cattle.
3. Of the human bone cases, 37.0% were classified as forensically relevant, having a PMI of 50 years or less. Evidence of peri-mortem trauma was present in 9.7% of the human bone finds.
4. In cases where the bones were matched to a missing person, identification was achieved by molecular genetic methods in 70.8% and by morphological methods in 29.7%.
5. Comparing the numbers with national studies did not reveal major differences. The results of comparable international studies strongly depend on the role of Legal Medicine and Forensic Anthropology in the respective country.
